# Robust and accessible in situ sequencing with an open-source automated sequencer

**DOI:** 10.64898/2026.10.18.759961

**Published:** 2026-10-23

**Authors:** Aixin Zhang, Zoe Maltzer, Jacob Lapka, Sabrina Cheng, Alan Guthrie, Cliff Slaughterbeck, Angela Y. Fan, Neha Goswami, Mara C.P. Rue, Maryam Majeed, Dennis M. Maharjan, Jeanelle Ariza, Angela P. Ayala, Madeleine Bixby, Cory T. Dickson, Samantha D. Hastings, Alvin Huang, Tulip E. O’Neill, Alana A. Oyama, Caleb Shi, Jack Waters, Yi-Chen Wu, Renae Galluccio, Huiqing Zhan, James A. Bourne, Gregory Horwitz, Justus M. Kebschull, Ian R. Wickersham, Peter Rudebeck, Alex Cahoon, Xiaoyin Chen

## Abstract

Targeted in situ sequencing underlies many spatial transcriptomic, barcoded connectomic, and multiplexed perturbation techniques. Each experiment requires many cycles of chemical reactions and imaging that are time-consuming and error-prone to perform manually, and few commercial platforms can sequence highly diverse RNA barcodes in situ at cellular resolution across large tissue sections. Here we present Seq-o-Matic, an open-source automated in situ sequencer built entirely from commercially available components, which integrates fluidics, heated incubation, and imaging of up to three continuous 18 mm × 50 mm areas on standard microscope slides. Relative to manual sequencing, Seq-o-Matic reduced hands-on time 6-to 17.5-fold and total run time about 2.6-fold. Across three runs spanning two mouse strains and a neonatal marmoset, sequencing quality, measured by signal retention, channel specificity, and phasing, was similar to that of four manually sequenced mouse brains. In four mouse hemispheres sequenced in separate runs (two C57BL/6J, one NOD, and one WSB), consensus cell types were present in every brain and corresponded to those of a manually sequenced C57BL/6J brain. Within cortical excitatory neurons, the two C57BL/6J brains were more similar to each other than were brains of different strains, suggesting that technical variation between runs was small relative to strain differences. Seq-o-Matic thus makes consistent, high-throughput in situ sequencing accessible to laboratories without specialized in situ sequencing expertise.

